# Compressive and Flexural Strength of 3D-Printed and Conventional Resins Designated for Interim Fixed Dental Prostheses: An In Vitro Comparison

**DOI:** 10.3390/ma15093075

**Published:** 2022-04-23

**Authors:** Mihaela Pantea, Robert Cătălin Ciocoiu, Maria Greabu, Alexandra Ripszky Totan, Marina Imre, Ana Maria Cristina Țâncu, Ruxandra Sfeatcu, Tudor Claudiu Spînu, Radu Ilinca, Alexandru Eugen Petre

**Affiliations:** 1 Department of Fixed Prosthodontics and Occlusology, Faculty of Dental Medicine, “Carol Davila” University of Medicine and Pharmacy, 17-23 Plevnei Street, 20221 Bucharest, Romania; mihaela.pantea@umfcd.ro (M.P.); tudor.spinu@umfcd.ro (T.C.S.); alexandru.petre@umfcd.ro (A.E.P.); 2 Department of Metallic Materials Science, Physical Metallurgy, University Politehnica of Bucharest, 313 Splaiul Independentei, J Building, 060042 Bucharest, Romania; ciocoiurobert@gmail.com; 3 Department of Biochemistry, Faculty of Dental Medicine, “Carol Davila” University of Medicine and Pharmacy, 17-23 Plevnei Street, 020021 Bucharest, Romania; maria.greabu@umfcd.ro (M.G.); alexandra.totan@umfcd.ro (A.R.T.); 4 Department of Complete Denture, Faculty of Dental Medicine, “Carol Davila” University of Medicine and Pharmacy, 17-23 Plevnei Street, 020221 Bucharest, Romania; marina.imre@umfcd.ro; 5 Department of Oral Health and Community Dentistry, Faculty of Dental Medicine, “Carol Davila” University of Medicine and Pharmacy, 17-23 Calea Plevnei Street, 010221 Bucharest, Romania; 6 Department of Biophysics, Faculty of Dental Medicine, “Carol Davila” University of Medicine and Pharmacy, 17-23 Calea Plevnei Street, 010221 Bucharest, Romania; radu.ilinca@umfcd.ro

**Keywords:** interim dental prosthesis, 3D printing, dental materials, polymers, mechanical tests, compressive strength, flexural strength

## Abstract

A provisionalization sequence is essential for obtaining a predictable final prosthetic outcome. An assessment of the mechanical behavior of interim prosthetic materials could orient clinicians towards selecting an appropriate material for each clinical case. The aim of this study was to comparatively evaluate the mechanical behavior—with compressive and three-point flexural tests—of certain 3D-printed and conventional resins used to obtain interim fixed dental prostheses. Four interim resin materials were investigated: two 3D-printed resins and two conventional resins (an auto-polymerized resin and a pressure/heat-cured acrylic resin). Cylindrically shaped samples (25 × 25 mm/diameter × height) were obtained for the compression tests and bar-shaped samples (80 × 20 × 5 mm/length × width × thickness) were produced for the flexural tests, observing the producers’ recommendations. The resulting 40 resin samples were subjected to mechanical tests using a universal testing machine. Additionally, a fractographic analysis of failed samples in bending was performed. The results showed that the additive manufactured samples exhibited higher elastic moduli (2.4 ± 0.02 GPa and 2.6 ± 0.18 GPa) than the conventional samples (1.3 ± 0.19 GPa and 1.3 ± 0.38 GPa), as well as a higher average bending strength (141 ± 17 MPa and 143 ± 15 MPa) when compared to the conventional samples (88 ± 10 MPa and 76 ± 7 MPa); the results also suggested that the materials were more homogenous when produced via additive manufacturing.

## 1. Introduction

The main functions of interim fixed prostheses in dental treatments can be summarized as follows: the protection and healing of dental, periodontal, and mucosal tissues; the facilitation and guidance of the healing process in peri-implant gingival tissue; temporization (periodontal splinting, space-maintainers, post-surgical prosthesis); the testing of certain parameters (aesthetics, new static and dynamic occlusal scheme, new vertical occlusal dimension, oral functionality); the evaluation of hygiene procedures; communication tool between dental team members; contribution to both patient comfort and confidence [1]. These types of restorations usually have a limited lifespan in prosthetic therapy; however, certain situations can be associated with their extended use (from a few weeks to more than one year), including complex oral rehabilitation cases; prosthetic treatments dedicated to children, adolescents, or elderly persons; the presence of certain systemic diseases that require the cessation or postponement of dental procedures; and the extension of the provisionalization sequence [1,2,3,4]. In addition to the above-mentioned aspects, the selection of the type of provisional fixed restorations must be adapted to each separate clinical case in a personalized manner, taking into consideration chewing forces (masticatory forces), bite force, chewing pattern, jaw muscle activity, parafunctions, diet, the age of edentulism, the length of edentulous spaces, and the type of prosthetic restoration (i.e., supported by dental implants or by natural teeth) [5,6]. As a consequence, the materials used for obtaining and cementing interim dental prostheses should exhibit a good biocompatibility and proper mechanical behavior [7,8,9]. The term biocompatibility “refers to the ability of a material to perform with an appropriate host response in a specific application”; it is one of the most important factors that controls the success of biomaterials [10]. In this regard, the quantity of monomer released into the oral cavity as a result of the incomplete polymerization of dental resins and their interaction with the oral environment (human gingival fibroblasts, osteoblasts, dental pulp cells, and macrophages) [7,8,9] represent issues of great importance in the domain of interim dental materials. On the other hand, interim fixed prostheses should demonstrate proper tensile, compressive, and flexural strength (especially for long-span interim fixed prostheses, which are required to resist failure from bending); wear resistance and hardness [11,12,13]; dimensional accuracy that is stable in time; and an acceptable level of repairability and color stability [14,15,16,17,18,19].

Conventional interim prosthetic materials can be divided into two groups according to their chemical composition [14,15]: materials based on monomethacrylates or acrylic resins and materials based on dimethacrylates or bis-acryl/composite resins, such as bisphenol A-glycidyl dimethacrylate and urethane dimethacrylate [14,15]. Moreover, interim prosthetic materials obtained by additive manufacturing seem to follow the same classification [14,15,20]; it should be noted, however, that the chemical composition of certain 3D-printed interim prosthetic materials has not been yet completely disclosed by the producers [14,20].

Interim fixed dental prostheses can be obtained via conventional direct techniques (chairside fabrication), indirect techniques (manufactured in the dental laboratory), or via mixed methods of indirect–direct provisionalization [2]. Over the decades, conventional self-cured and pressure-cured acrylic resins have been frequently used in the direct, indirect, or indirect–direct manufacture of interim dental prostheses due to their affordability, favorable working characteristics, polishability, and easy manipulation and repair [1,3,9,11,12,19]. However, improved conventional interim prosthetic materials have been introduced relatively recently, such as bis-glycidyl methacrylate and bis-acryl-based materials with better aesthetics, better mechanical properties, and lower polymerization shrinkage than acrylic resins [7,14,15]. More recent technologies, such as additive manufacturing (AM; 3D printing) and subtractive technology (milling), represent indirect modern CAD/CAM (computer-aided design/computer-aided manufacturing) methods for obtaining interim dental prostheses [21,22,23]. In particular, 3D printing in dentistry usually employs five different printing technologies: stereolithography (SLA), digital light processing (DLP), selective laser melting (SLM), selective laser sintering (SLS), and fused deposition modeling (FDM) [24]. Although all these 3D printing technologies have different weaknesses and strengths [25], additive manufacturing is enthusiastically embraced by dental professionals as it offers various advantages when compared to traditional manufacturing, including reduced production time (speed), less material waste, lower costs, easy mass customization, the independence of the milling instruments, the combination of materials, higher quality, and innovation/transformation [26,27,28].

These aspects relating to the particularities of interim dental prosthetic materials represent subjects of interest in present-day medical scientific research and, at the same time, require continuous evaluation [14,15]. Particularly with regard to the mechanical behavior of interim prosthetic materials, the scientific literature [2,11,29] underlines that, in general, modern interim prosthetic materials (such as milled and 3D-printed resins obtained using CAD/CAM technology) are more reliable for clinical applications than the conventionally polymerized materials [2,11,29]. Apart from this, other scientific studies [30,31,32] have shown that CAD/CAM milled and 3D-printed resins can ensure a higher accuracy compared to the conventional materials.

Concerning the additive manufacture of interim prostheses, the scientific dental literature offers relatively scarce information regarding the effect of different printing parameters and post-polymerization procedures on the printing accuracy, superficial roughness, and mechanical properties of the 3D-printed resins [5]. In one study, it was suggested [28] that the printing orientation should be adapted to the different load directions in the mouth (i.e., the bite force is preferably applied vertically for a molar, while a canine tooth is more exposed to transverse loadings) [28]. In the same line, another study [5] showed that vertically 3D-printed prosthetic restorations, where the layer orientation was perpendicular to the loading direction, presented a significantly higher compressive strength than horizontally printed restorations [5]. On the other hand, the flexural strength of 3D-printed interim fixed dental prostheses was found to be higher [29] when the build direction was set at a 30° orientation than when it was set at 0° (the occlusal surface of the restoration was facing downwards), or 45°, 60°, and 90° (the occlusal surface of the restoration was facing the lateral side). In a previous study [29], the mean values of flexural strength for the tested three-unit interim prostheses were as follows: 1053 ± 168 N for a build direction of 0°; 1183 ± 188 N for a build direction of 30°; 1178 ± 81 N for a build direction of 45°; 1166 ± 133 N for a build direction of 60°; and 949 ± 170 N for a build direction of 90° [29]. Moreover, different post-polymerization procedures can significantly influence the fracture resistance of 3D-printed interim restorations [12] and artificial aging significantly decreases the fracture resistance of these types of restorations [5,11,13,18].

We are currently witnessing the extensive development, happening at an unprecedented pace, of biomaterials and technologies related to 3D printing in the medical and pharmaceutical fields, which have applications in tissue regeneration and cancer investigations [33]. These applications also address the oral and maxillofacial region. In addition, 3D printing facilitates workflow in dentistry and builds a sustainable dental healthcare system [34].

The assessment of the diverse mechanical properties of modern 3D-printed interim prosthetic materials could contribute to a better understanding of their behavior in oral conditions, thus enabling dental practitioners to choose the most appropriate interim prosthetic material for each separate clinical case [34,35]. Furthermore, any mechanical problems of interim prosthetic restorations may cause discomfort for patients, as well as financial and economic loss [12].

Given this context, the purpose of the present study was to investigate the mechanical behavior (compressive and flexural strength) of interim prosthetic resins obtained via conventional methods in comparison to additive manufactured ones. Additionally, a fractographic analysis of samples that failed in bending was performed in order to highlight aspects related to the structural homogeneity of the materials included in the study.

## 2. Materials and Methods

### 2.1. Fabrication of Resin Samples

The following dental materials used in the manufacture of interim fixed dental prostheses were selected to be tested during this study: NextDent C&B MFH, NextDent by 3D Systems, Vertex B.V., Soesterberg, The Netherlands (a 3D-printed resin that must be used in combination with laser or DLP (digital light processing)-based 3D printers that support NextDent resins); HARZ Labs Dental Sand, color A3, HARZ Labs, Riga, Latvia (a 3D-printed resin designated for desktop LCD (liquid crystal display) printers); Duracyl, SpofaDental a.s., Jicin, Czech Republic, KaVo Kerr Group (an auto-polymerized/self-cured conventional acrylic resin); and Superpont C+B, SpofaDental a.s., Jicin, Czech Republic, KaVo Kerr Group (a pressure/heat-cured acrylic resin). Our study included both modern resins used for manufacturing interim fixed dental prostheses (3D-printed resins) and conventional resins (auto-polymerized and pressure/heat-cured acrylic resins). Moreover, the NextDent C&B MFH resin was produced using a proprietary additive manufacturing system, while the 3D-printed HARZ Labs Dental Sand could be manufactured via an open processing route. The main criteria for the selection of the dental materials investigated in this study included the following aspects: the materials should be intended for the manufacturing of interim dental prostheses; the materials should allow the fabrication of interim dental prostheses for both short- and long-term temporary use; the resins should be commercially available; and the materials should be acknowledged for their use in interim prosthetic therapy.

The name, manufacturer, material type, and chemical composition of the investigated materials are summarized in Table 1. The details were provided by the manufactures.

Cylindrically shaped samples (25 × 25 mm/diameter × height) were designed for the compression tests, while bar-shaped samples (80 × 20 × 5 mm/length × width × thickness) were designed for the flexural tests. For each material, five samples were fabricated for each type of test, resulting in a total of 40 samples.

The 3D-printed samples were manufactured using CAD/CAM (computer-aided design/computer-aided manufacturing) technology. EXOCAD 2.2 Valetta was used as a CAD software for the 3D-printed NextDent C&B MFH samples, while the 3D rendering was performed using the CAM software 3D SPRINT from 3D Systems, Rock Hill, SC, USA. STL files were generated and exported to a 3D printer (NextDent 5100, NextDent by 3D Systems, Vertex B.V., Soesterberg, The Netherlands) in order to fabricate the printed resin samples. The software licenses are currently available. The bottle containing the printable resin was manually shaken for at least 5 min prior to initiating the mixing process on the NextDent LC-3DMixer (NextDent by 3D Systems, Vertex B.V., Soesterberg, The Netherlands), which lasted one hour. The printer reservoir was filled with the mixed resin and the samples were printed according to the manufacturer’s recommendations. The printing parameters were automatically set—the printer adjusted them by scanning the QR code on the printable liquid resin container. The print resolution was set at 50 μm.

When the printing program ended, the building platform was removed from the machine and placed on a clean cloth, with the samples facing upwards. The printed samples were removed from the platform and cleaned for three minutes in ethanol (96%), in order to remove any excess resin, using an ultrasonic bath, after which they underwent another cleaning process for two minutes in clean ethanol (96%). After drying, the printed samples rested for at least 10 min in order to ensure that they were free of any ethanol residues. Afterwards, the printed parts were placed in a UV light curing box for 30 min for the final, optimal polymerization (NextDent LC-3DPrint Box, NextDent by 3D Systems, Vertex B.V., Soesterberg, The Netherlands) at a curing temperature of min. 60 °C with the recommended wavelength of blue UV-A 315–400 nm. Subsequently, the support structures were removed, and the samples were finished and polished manually.

The 3D-printed samples obtained from HARZ Labs Dental Sand resin were designed using the CHITUBOX software. Thus, the resulting STL file was imported to CHITUBOX V1.65. The 3D printer (Phrozen Sonic Mini 4K 3D printer, Phrozen Technology, Xiangshan Dist., Hsinchu, Taiwan) was connected to the computer and the default support generation parameters were set. The 3D file was saved as a CTB file, then imported to a USB. The building platform and resin vat were inserted into the printer. The bottle containing the printable resin was manually shaken for one minute, then the resin was poured into the printer reservoir up to the mark on the resin vat. The USB was inserted into the printer and the 3D printing process was started. Once the printing was complete, the building platform was removed from the printer and placed onto a table, on a clean cloth. The printed samples were removed from the platform with a metal scraper and cleaned for three minutes in 96% ethanol. Afterwards, the samples were cured in a post-curing chamber (Form Cure Formlabs, Formlabs Inc., Somerville, MA, USA) for 30 min. The resulting 3D-printed samples were finished and polished manually.

The auto-polymerized acrylic resin samples (Duracyl, SpofaDental a.s., Jicin, Czech Republic, KaVo Kerr Group) were obtained by the means of silicone patterns (high consistency laboratory condensation silicone, Zetalabor Rigid C-Silicone Lab Putty, Zhermack, Badia Polesine (RO), Italy). The 3D-printed samples manufactured from NextDent C&B MFH resin were indented with this type of silicone to obtain a high consistency silicone pattern composed of two parts (lower and upper—similar to a lid), which allowed the fabrication of the cylindrical and bar-shaped samples with the above-mentioned dimensions. A separating solution—Isodent (SpofaDental a.s., Jicin, Czech Republic, KaVo Kerr Group)—was applied inside the silicone conformers with the aim of ensuring a smooth detachment of the resin samples. The powder and the monomer were mixed according to the manufacturer’s instructions; the mixing ratio was 2 g of powder and 1 g of liquid, which represents a volume ratio value of about 3:1 (3 parts powder to 1 part liquid). The powder was added to the liquid and the mixture was prepared in a glass container. The resulting paste was mixed for about 30 s at room temperature (23 ± 2 °C) with a stainless steel spatula until a homogenous consistency was obtained. Bars and cylinders with the same forms and dimensions as the 3D-printed samples were fabricated by dispensing the material in the silicon pattern. The mixture, while still pasty, was inserted into the lower silicone pattern using appropriate dental instruments (dental composite non-stick spatula and pluggers). Upon the insertion and levelling of the resin, the upper part of the silicone pattern was slightly pressed against the lower part in order to eliminate the excess material and obtain an external surface that was as flat as possible. The material was allowed to cure observing the manufacturer’s recommendations; the curing lasted for about 10–12 min at room temperature, 23 ± 2 °C.

In order to fabricate the pressure/heat-cured acrylic resin samples (Superpont C+B, SpofaDental a.s., Jicin, Czech Republic, KaVo Kerr Group), silicone patterns (high consistency laboratory condensation silicone, Zetalabor Rigid C-Silicone Lab Putty, Zhermack, Badia Polesine (RO), Italy) were obtained for each of the two types of samples (cylindrical and bar-shaped), as previously described. The silicone patterns were isolated using a separating solution—Isodent (SpofaDental a.s., Jicin, Czech Republic, KaVo Kerr Group). A resin paste was obtained by mixing the powder with the appropriate quantity of liquid/monomer (in a volume ratio of 3:1, i.e., 3 parts powder to 1 part liquid) in a glass container. The mixture was thoroughly blended for about 30 s at room temperature (23 ± 2 °C) with a stainless-steel spatula until a homogenous consistency was obtained. The resulting paste was pressed into the silicone conformers. The two parts of the conformers were closed and manually pressed, slowly and progressively, so that the paste thoroughly filled the patterns, and the excess mixture was removed. The samples were maintained at 93 °C under 0.6 MPa pressure for 25 min in order to achieve final polymerization. When the curing process was completed, the samples were allowed to cool and were removed from the pressure unit.

After manufacturing, all samples were finished and polished manually using a magnifying lamp × 3 (Magnifying lamp LC101LED, Nuova A.S.A.V., Cavriago (RE), Italy). The samples were stored in a closed box at room temperature (23 ± 2 °C) and 50 ± 5% relative humidity for 3 days prior to performing the mechanical tests. During this time, the samples were assigned codes, marked, and measured. The measurements were performed using a digital caliper (OEM Tools 25363, OEM Tools, New York, NY, USA) to verify the length, width, and thickness of the bar-shaped samples and the diameter and height of the cylindrical samples. Three measurements were made within different sections along the samples for each dimension (length, width, and thickness for bar-shaped samples and diameter and height for cylindrical specimens).

The following codes were assigned to the resin samples:

- 3DCS: The 3D-printed resin samples made from NextDent C&B MFH, NextDent by 3D Systems, Vertex B.V., Soesterberg, The Netherlands.
- 3DOS: The 3D-printed resin samples made from HARZ Labs Dental Sand, HARZ Labs, Riga, Latvia;
- CAP: The conventional auto-polymerized resin samples made from Duracyl, SpofaDental a.s., Jicin, Czech Republic, KaVo Kerr Group;
- CHP: The conventional pressure/heat-cured acrylic resin samples made from Superpont C+B, SpofaDental a.s., Jicin, Czech Republic, KaVo Kerr Group.

### 2.2. Mechanical Tests

The goal of this experiment was to investigate the mechanical behavior of the interim resin samples prepared via conventional methods in comparison to those produced using additive manufacture. Since no specific standards are available for additive manufactured products, the compression tests were performed in accordance with ASTM (American Society for Testing and Materials) D695 “Standard test method for compressive properties of rigid plastics”, while the flexural tests were carried out in accordance with ASTM D790 “Standard test methods for flexural properties of unreinforced and reinforced plastics and insulating materials”. We chose these procedures in order to attain, to some degree, a common denominator for the tests performed, in a reproducible and repeatable manner, on samples obtained by completely distinct fabrication methods.

#### 2.2.1. Compression Tests

**Figure 1:**
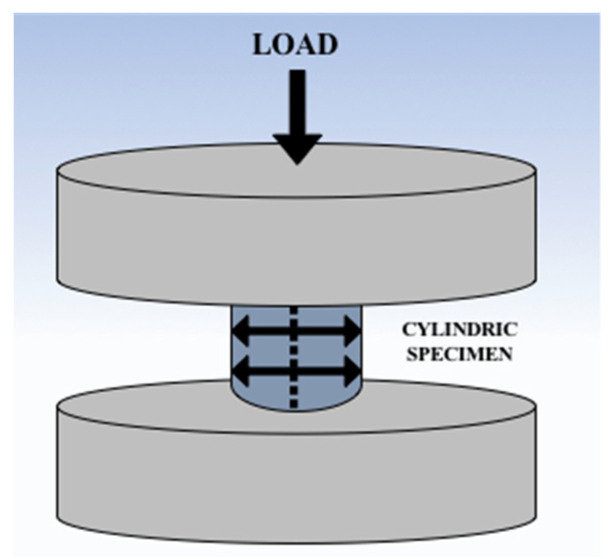
Compression test setup with steel platens and resin specimen.

Cylindrically shaped test samples were prepared from each material and subjected to compression testing by fitting them on the universal testing machine (Walter + Bai LFV 300, Walter + Bai AG, Löhningen, Switzerland). The compression tool consisted of two platens made of hardened steel that ensured axial loading, and the test speed was set at 5 mm/min. A schematic illustration of the compressive tests is presented in Figure 1.

#### 2.2.2. Flexural Tests (Three-Point Bending Tests)

**Figure 2:**
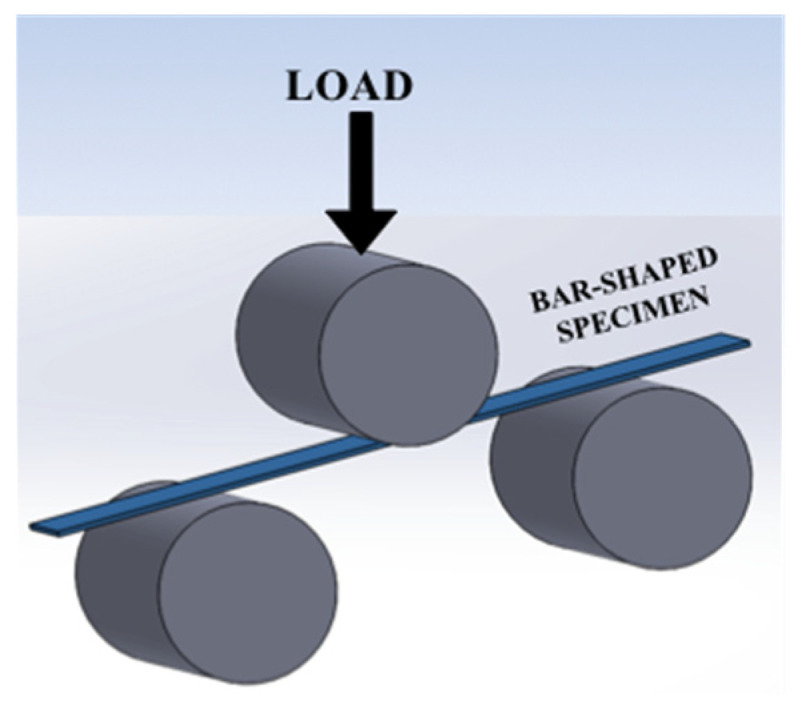
Schematic depicting three-point bending fixture and test specimen.

The flexural tests were performed using a three-point bending configuration by mounting a dedicated tool on the universal testing machine (Walter + Bai LFV 300, Walter + Bai AG, Löhningen, Switzerland). The radius of the support rollers and the loading nose was 25 mm and the distance between the centers of the rollers was 60 mm. The test samples were prismatic (bar-shaped samples), and each testing set comprised 5 specimens. The test speed was kept constant at 5 mm/min. A schematic illustration of the three-point bending tests is shown in Figure 2. The mechanical tests were conducted at room temperature (23 ± 2 °C) and 50 ± 5% relative humidity.

#### 2.2.3. Fractographic Analysis of Samples That Failed the Flexural Tests

Upon finalizing the three-point bending flexural tests, the fracture surfaces of the bar-shaped resin samples were subjected to a fractographic analysis that was performed by employing a portable digital stereomicroscope for image acquisition and the AxioVision software for image editing.

## 3. Results

The experimental part of this study comprised compression and three-point bending tests performed on polymers/resins used in dentistry for obtaining interim fixed dental prostheses. The CAP and CHP samples were produced via conventional procedures used in dentistry, while the 3DCS and 3DOS samples were obtained by employing the additive manufacturing processes, as previously described. The geometry of the test samples was consistent with the standard specifications and each testing set comprised five samples. The compression and flexural tests were carried out in accordance with the ASTM, as mentioned above. The testing parameters were determined according to the standard specifications and sample dimensions (where required).

The mechanical behavior was evaluated using the stress-strain curves associated with sample behavior during the test in addition to several parameters. During the compression tests, the elastic modulus, proportionality limit, and yield strength were determined by processing the stress-strain data. Afterwards, sample shortening and barreling were measured on the tested samples. On the other hand, using the data acquired during the flexural tests, the elastic modulus, flexural strength, and test sample rigidity were determined. The analysis of the surfaces where fractures occurred during the flexure tests was performed using a digital stereomicroscope.

### 3.1. Compression Tests

**Figure 3:**
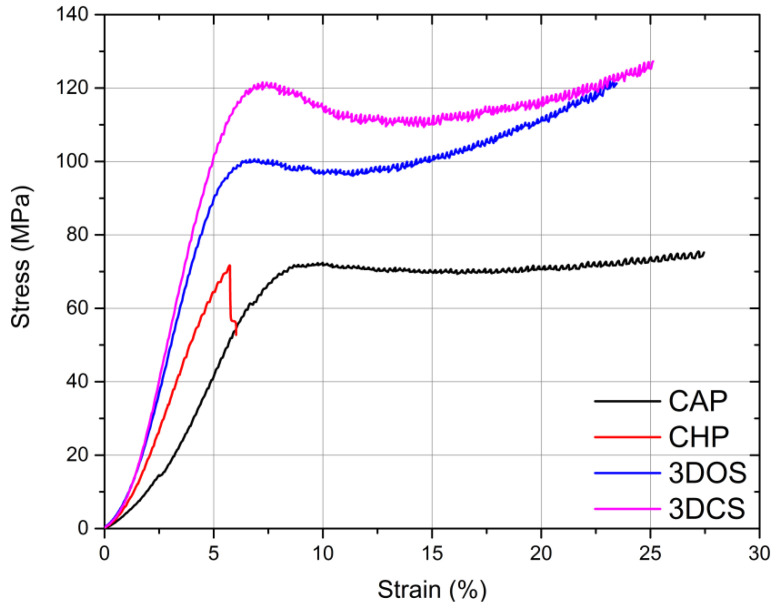
Representative stress-strain curves in compression.

The compression test was stopped when a specified displacement was reached (common to all tests) or when the sample failed prior to reaching the envisaged value. The test data were acquired by way of the universal machine proprietary software and were post-processed using graphing and mathematical software (ORIGIN 2016, OriginLab Corporation, Northampton, MA, USA and Mathematica 11.3, Wolfram Research, Long Hanboroug, Oxfordshire, UK). Representative stress-strain curves corresponding to the compression tests are presented in Figure 3.

The behavior observed during compression revealed that the materials used for fabricating the CAP, 3DOS, and 3DCS samples were malleable, given the amount of plastic deformation, while the material with which the CHP samples were made failed in a brittle manner (almost no plastic deformation). All malleable samples were deformed by barreling, while the samples in the CHP set failed by forming cracks parallel to the loading direction.

**Figure 4:**
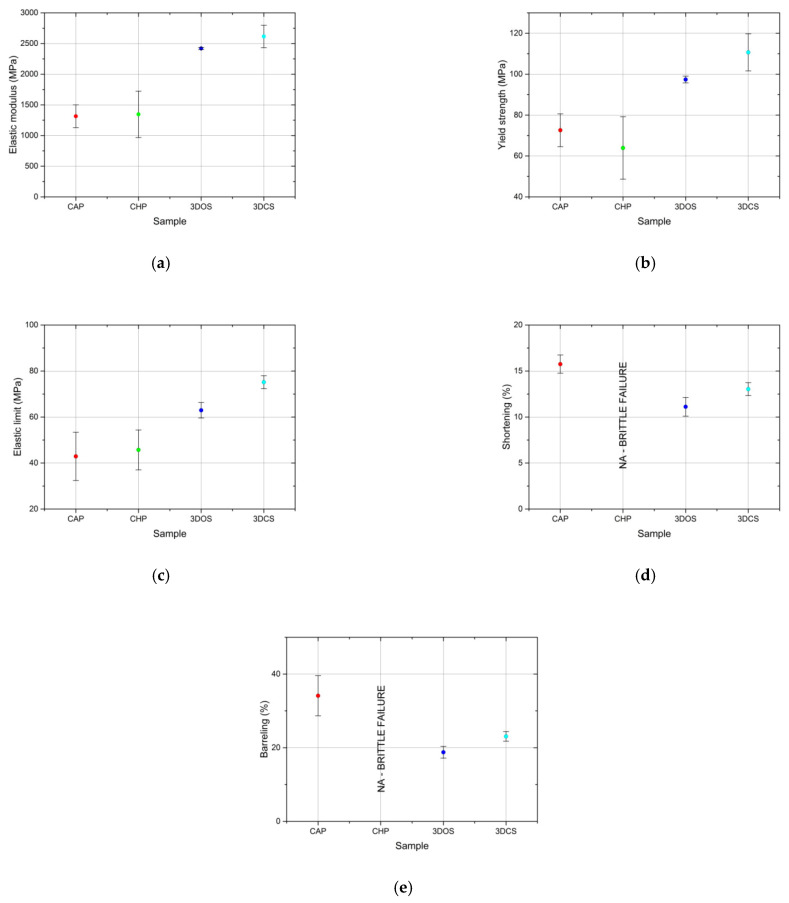
Comparison of average values for (**a**) elastic modulus, (**b**) yield strength, (**c**) elastic limit, (**d**) sample shortening, and (**e**) barreling of the materials tested in compression.

Given the malleable behavior of the CAP, 3DOS, and 3DCS materials, a value for the compressive strength could not be calculated; instead, a strength parameter equivalent to the compressive strength was determined as the yield strength. This parameter was calculated using the same procedure as tensile testing for the determination of the conventional yield strength, by offsetting with 0.2% strain. The offsetting was performed post curves’ nose compensation by disregarding the incipient region of the stress-strain curve where it was assumed that the material and compression tool were settling. The determined parameters are presented in Table 2 and the results are presented graphically in Figure 4a–e.

The elastic modulus values corresponding to the additive manufactured samples fell into the same range—2.4 ± 0.02 GPa for 3DOS and 2.6 ± 0.18 GPa for 3DCS—and were considerably higher than the values obtained for conventionally manufactured samples, with 1.3 ± 0.19 GPa for CAP and 1.3 ± 0.38 GPa for CHP (as seen in Figure 4a).

A comparison of the average yield strength values of the tested materials is depicted in Figure 4b. The additive manufactured samples demonstrated, once again, better results than the conventionally manufactured specimens; the samples in the 3DCS set obtained the highest value (110 ± 9 MPa), followed by the 3DOS set (97 ± 2 MPa). The conventional fabrication methods generated materials with lower mechanical characteristics, where the lowest values corresponded to the CHP set (64 ± 15 MPa; actually the compressive strength), preceded by the CAP set (73 ± 8 MPa).

The elastic limit is considered to be the point (stress value) where the linear relationship between stress and strain no longer holds. By this definition, the elastic limit is equivalent with the proportionality limit; this assumption is currently applied in practice given the difficulties in accurately determining these two distinct values. Regarding this strength parameter, the additive manufactured samples again exhibited considerably higher values (75 ± 3 MPa for 3DCS and 63 ± 3 MPA for 3DOS) than the conventionally fabricated samples (43 ± 10 MPa for CAP and 46 ± 9 MPa for CHP). In the latter category, the lowest value was recorded for the CAP samples (43 ± 10 MPa), while the highest corresponded to the 3DCS samples (75 ± 3 MPa; Figure 4c).

**Figure 5:**
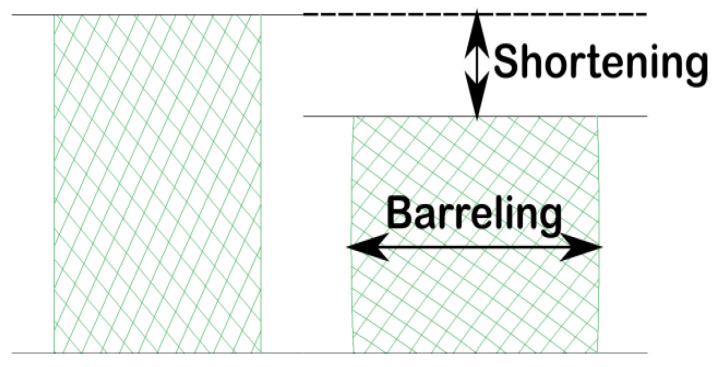
Schematic illustration of shortening and barreling.

Sample shortening (Figure 5) is determined as the variation in sample height (initial height less post-test height) divided by the initial sample height and is considered to be a descriptor for the malleability of a material. Although it is a parameter used for evaluating the characteristics of metallic materials, in this particular case, we treated it as a descriptor of the mechanical behavior of the materials, along with sample barreling. Since the masticatory forces manifesting inside the oral cavity vary, and various incidents, such as accidentally biting hard objects, are likely to occur, materials that are capable of deformation are preferred to materials that will shatter. The additive manufactured samples, 3DCS and 3DOS, appeared to have lower shortenings (13 ± 0.7% for 3DCS and 11 ± 1.0% for 3DOS) than the conventional material CAP (16 ± 1.0%). The CHP material failed in a brittle manner; therefore, its shortening could not be determined (Figure 4d).

Similar to sample shortening, sample barreling can be regarded as a descriptor for material malleability; it is determined as the variation of the transverse area of the test sample (post-test transverse area less initial transverse area) divided by the initial transverse area. The samples in the CHP set failed in a brittle manner and no barreling could be determined, while the sample sets obtained by additive manufacturing exhibited less barreling (23 ± 1.0% for 3DCS and 19 ± 1.6% for 3DOS) than those in the CAP set (34 ± 5.0%; Figure 4e). During the compression tests, the additive manufactured samples demonstrated a more adequate mechanical behavior than the specimens obtained by conventional processing methods. As 3D-printed resins are more rigid, they tend to deform less when loading is being applied in the elastic region (the normal masticatory forces induce loading in the elastic region); thus, it is less likely for the patient to feel a “slack” in the interim restoration during mastication. The higher values of the strength parameters (elastic limit and yield strength) of the additive manufactured samples indicate that they can withstand accidental overloads. At the same time, their ability to undergo plastic deformation can also be regarded as an advantage; instead of sudden (brittle) failure in the case of an overload, they are capable of absorbing energy and deforming prior to failure.

### 3.2. Flexural Tests

The data obtained during the three-point bending tests (flexural tests) were processed using graphing and mathematical software (ORIGIN 2016, OriginLab Corporation, Northampton, MA, USA and Mathematica 11.3, Wolfram Research, Long Hanboroug, Oxfordshire, UK) with the aim of determining the strength parameters of the materials, which are presented in Table 3.

**Figure 6:**
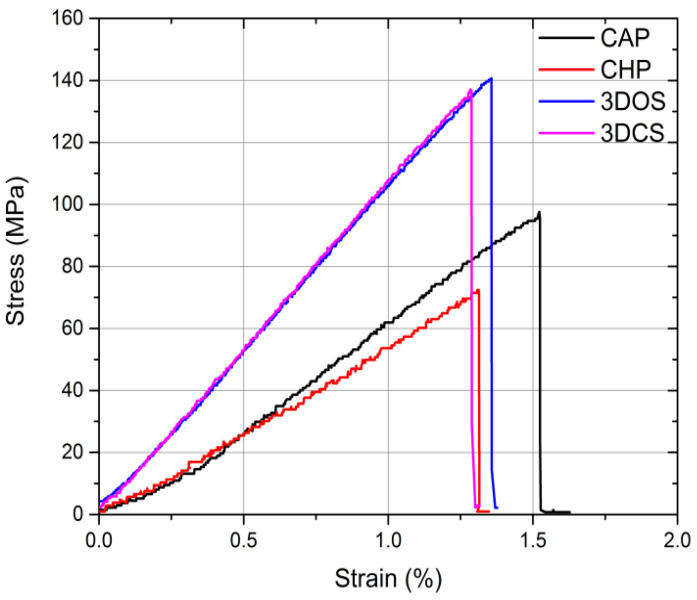
Representative stress-strain curves in bending.

An example of stress-strain curves in bending is depicted in Figure 6.

**Figure 7:**
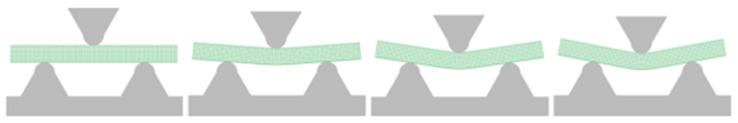
Schematic of the behavior of the samples during three-point bending tests.

All samples failed the flexural tests (a mixed type of loading) without a significant amount of plastic deformation, manifesting a brittle mechanical behavior. Another important aspect to be mentioned is that the additive manufactured samples (3DOS and 3DCS) were more rigid than the conventionally fabricated samples. A graphic representation of the behavior of the samples during the three-point bending tests is shown in Figure 7.

**Figure 8:**
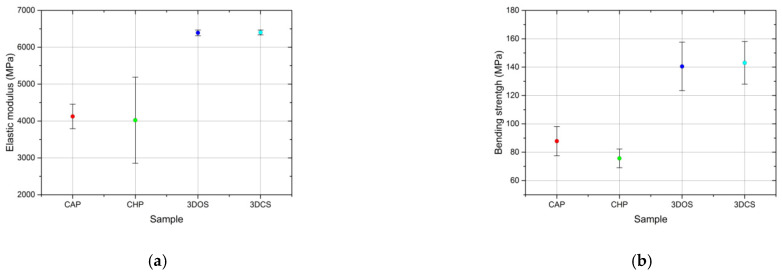

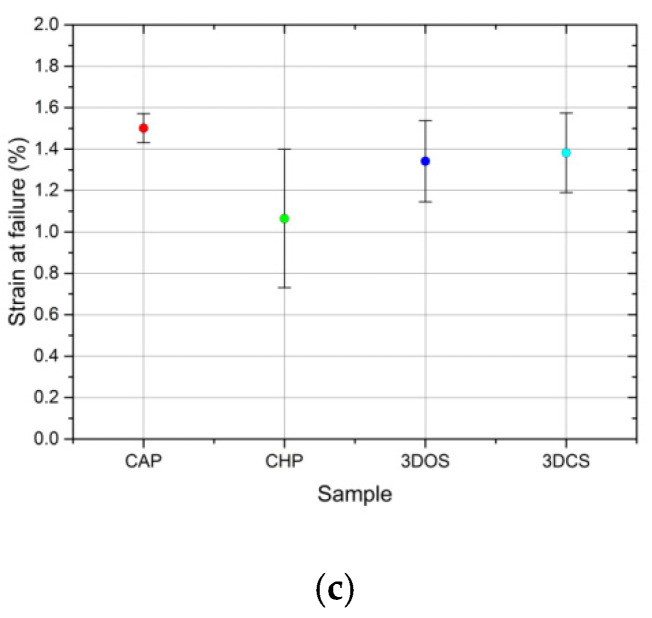
Comparison of average values for (**a**) elastic modulus, (**b**) bending strength, and (**c**) strain at failure of the materials tested in bending.

Based on the data obtained, the strength parameters, i.e., the elastic modulus in bending and bending strength, were determined, along with the strain at failure, which is considered to be a descriptor of the plasticity and rigidity of an assembly. A comparison of the average values of the elastic modulus in bending is shown in Figure 8a–c.

The additive manufactured samples showed higher elastic moduli (6402 ± 69 MPa for 3DCS and 6329 ± 79 MPa for 3DOS) than the conventionally fabricated samples (4124 ± 333 MPa for CAP and 4022 ± 1167 MPa for CHP; Figure 8a). The standard deviation is a measurement of the spread of data that can be mainly attributed to material inhomogeneity and random errors that occur during the test. Here, the impact/weight of the random errors can be considered negligible since all samples were subjected to the same testing procedure. Therefore, it was concluded that the data spread could be mostly attributed to material inhomogeneity. Thus, in contrast with the additive manufacturing process, the conventional fabrication procedures generate inhomogeneous products, and the repeatability and reproducibility could be strongly dependent upon the technician’s skills.

The bending strength comparison presented in Figure 8b indicates a tendency similar to that observed for the elastic modulus in bending. Once more, the additive manufactured samples showed a higher bending strength (143 ± 15 MPa for 3DCS and 141 ± 17 MPa for 3DOS) than the conventionally fabricated specimens (88 ± 10 MPa for CAP and 76 ± 7 MPa for CHP).

Strain at failure represents the strain corresponding to the stress where failure occurs and can be easily identified on the stress-strain curves in Figure 6 as a sudden drop in the stress. A comparison of the average values of the strain at failure is presented in Figure 8c; all samples showed similar values, falling within the range of 1.0–1.6%.

Strain at failure can be regarded as a descriptor for the plasticity of the material, i.e., its ability to deform prior to failure. All tested materials showed similar values for strain at failure, which indicated that they were unable to undergo plastic deformation prior to failure in bending. Hence, we concluded that the results were mainly generated by the poor tensile mechanical behavior (brittle).

### 3.3. Fractographic Analysis of Samples That Failed the Flexure Tests

Given that relatively scarce information regarding the material characteristics and type for the polymers (especially for the 3D-printed samples) tested in this study is available, a fractographic analysis of the failed samples in bending proved to be difficult; however it was deemed to be helpful.

Fractography studies with naked eye investigations and the use of a stereomicroscope offer, in the case of polymers, relevant information on the cause of failure and loading type; such an analysis, apart from being quickly and easily performed in situ, could provide more information than the current high-performance and high magnification investigation techniques (i.e., scanning electron microscopy and high resolution scanning electron microscopy). The fractographic analysis was performed by employing a digital portable stereomicroscope for image acquisition and the AxioVision software (AxioVison, Carl Zeiss Ltd., Cambridge, UK) for image editing, as previously mentioned.

**Figure 9:**
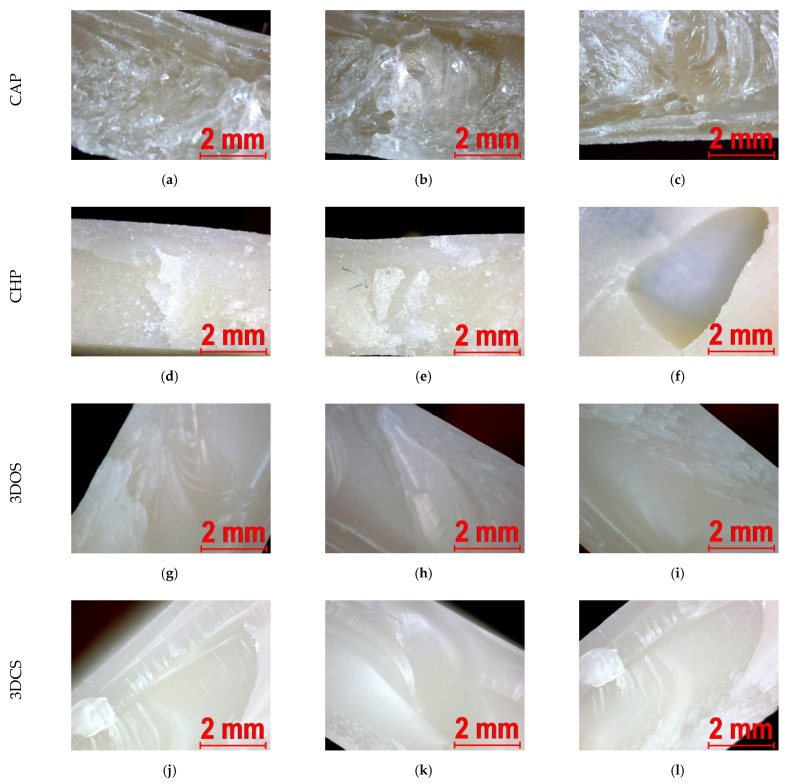
Fracture surfaces of resin samples failed in bending: (**a**–**c**)—images corresponding to CAP samples; (**d**–**f**)—images corresponding to CHP samples; (**g**–**i**)—images corresponding to 3DOS samples; (**j**–**l**)—images corresponding to 3DCS samples.

The material properties, polymer structure, stress state, geometry, and environment dictate to a large extent the appearance of the fracture surface. In this particular case, almost all variables were known, except for the structure (at a macroscopic level), which was conditional upon the production method. As shown in Figure 9, the fracture surfaces, regardless of the production method, displayed a smooth and glossy appearance that was associated with a brittle fracture, as observed during flexural testing.

Another feature that was considered was surface whitening, clearly visible in Figure 9a–f, which occurred in polymers when they were stressed beyond their yield strength, usually located near or at the end of the failure. High stress concentrations appeared as defects in the material, with Figure 9c,f showing strong whitening in the proximity of voids. Curved striations were observed on all fractured surfaces, having occurred during a fast brittle fracture, and were be associated with crack propagation or arrest in the material, thus steps emerged on the surface.

Bending is a mixed type of loading involving a tensile and compressive component. Most of the time, the fracture origin was found on the surface subjected to maximum tensile stress (on the surface opposite to the loading nose of the fixture), and in the case of CHP resins, it was sometimes located inside the sample, at voids. In the proximity of the fracture origin, a so-called mirror zone (a surface that was smooth, flat, and reflected light) was observed (Figure 9a,k), followed by rib markings, which were strong curved markings (Figure 9g,k) with random spacing. Ratchet marks were mostly noted on additive manufactured samples (Figure 9g–l), with stress whitening.

The structure (at a macroscopic level) of additive manufactured samples is different, as the layers are added in succession; thus, a more fine-decorated fracture surface is expected to emerge when compared to conventionally fabricated samples, obtained using bulk material.

## 4. Discussion

The results of the present study indicated that the 3D-printed resin in the 3DCS set (NextDent C&B MFH, NextDent by 3D Systems, Vertex B.V., Soesterberg, The Netherlands) demonstrated the best mechanical behavior during the compression tests (yield strength: 110 ± 9 MPa; average elastic modulus values: 2615 ± 183 MPa). Consequently, this material (3DCS) manifested the best malleability, resilience, and toughness, absorbing a higher quantity of energy until reaching the breaking point, a detail that is important especially when masticatory forces are abruptly applied on the prosthetic restorations. The second best results corresponded to the 3DOS 3D-printed resin (HARZ Labs Dental Sand, HARZ Labs, Riga, Latvia; yield strength: 97 ± 2 MPa; average elastic modulus value: 2419 ± 16 MPa), followed by the values obtained for the auto-polymerized acrylic resin/CAP (Duracyl, SpofaDental a.s., Jicin, Czech Republic, KaVo Kerr Group; yield strength: 73 ± 8 MPa; average elastic modulus value: 1315 ± 186 MPa) and the pressure/heat-cured acrylic resin/CHP (Superpont C + B, SpofaDental a.s., Jicin, Czech Republic, KaVo Kerr Group; yield strength: 64 ± 15 MPa; average elastic modulus value: 1346 ± 379 MPa). The mechanical behavior in compression observed for the conventional resins (CAP and CHP) was characterized as being fragile, inhomogenous, and failing without absorbing energy.

Concerning the results of the flexural tests, the following succession was observed: the highest values were obtained for the 3DCS material (NextDent C&B MFH, NextDent by 3D Systems, Vertex B.V., Soesterberg, The Netherlands; elastic modulus: 6402 ± 69 MPa, average bending strength value: 143 ± 15 MPa), followed by the other resins (3DOS, CAP, and CHP), which demonstrated similar mechanical behavior and characteristics in bending with slight differences (elastic modulus: 6329 ± 79 MPa for 3DOS, 4124 ± 333 MPa for CAP, and 4022 ± 1167 MPa for CHP; average bending strength values: 141 ± 17 MPa for 3DOS, 88 ± 10 MPa for CAP, and 76 ± 7 MPa for CHP). It was noted that, in the case of the three-point bending tests, all the materials manifested fragility.

The masticatory process and the bite force are influenced by diverse factors, such as the number of the existing teeth, dental and periodontal status, the number and stability of occlusal contacts, jaw muscle activity, age, the presence of dental prostheses (fixed or removable; supported on implants or natural teeth), and temporomandibular disorders [35,36]. As an example, a previous study [36] indicated that maximum voluntary bite force (MBF) varies depending on age and sex; the average value of maximum voluntary bite force for females was 33–51 kgf and for males was 42–63 kgf, and a stronger bite was recorded for younger subjects than for older ones. This study [36] was conducted on a sample composed of 426 subjects (213 females and 213 males), aged from 20 to over 79 years. It was reported that the greatest masticatory force was required to penetrate rye bread (167 N = 17 kgf), followed by raw carrot (118 N = 12 kgf), boiled meat (80 N = 8 kgf), raw cabbage (74 N = 7.5 kgf), and cooked meat (124 N = 12.5 kgf) [36,37]. In normal conditions, the medium masticatory force varies between 70–200 N (although its value can reach 500–700 N or more), and only 40% of the MBF (maximum voluntary bite force) represents the real masticatory force [1,36,37]. With regard to our results, considering the compressive strength as equal to the yield strength (approximatively 73 MPa for CAP, 64 MPa for CHP, 97 MPa for 3DOS, and 110 MPa for 3DCS) and using a usual masticatory force of 200 N, we estimated the area needed to withstand this force, namely 4.65 mm^2^ for CAP, 4.35 mm^2^ CHP, 3.17 mm^2^ for 3DOS, and 2.67 mm^2^ for 3DCS. For a force of 700 N, the surfaces would be 16.28 mm^2^ for CAP, 15.27 mm^2^ CHP, 11.11 mm^2^ for 3DOS, and 9.33 mm^2^ for 3DCS. An increase in compressive strength leads to a decrease in the useful section needed to withstand the applied forces; thus, it can be said that interim prosthetic restorations with thinner walls or finer shapes can be made from materials with such characteristics.

On the other hand, it is worth noting that in our study, the obtained average value of flexural strength for the 3D-printed resin coded 3DCS (NextDent C&B MFH, NextDent by 3D Systems, Vertex B.V., Soesterberg, The Netherlands) was 143 ± 15 MPa, which was higher when compared to the value provided by the manufacturer: 85 MPa [29]. Additionally, the obtained average values of flexural strength for the conventional resins corresponded to the values provided by the manufacturers. Thus, for the pressure/heat-cured acrylic resin coded CHP (Superpont C + B, SpofaDental a.s., Jicin, Czech Republic, KaVo Kerr Group), the obtained average value for flexural strength was 76 ± 7 MPa, and that provided by the manufacturer was ≥50 MPa; for the auto-polymerized resin, the obtained average value for flexural strength was 88 ± 10 MPa, and that provided by the manufacturer was ≥65.5 MPa. Therefore, all the materials tested in our study showed appropriate mechanical behavior, withstanding forces comparable to those acting during the physiological masticatory process.

The results of our study are generally in line with the information found in the dental literature, where certain studies highlight the qualities of 3D-printed interim resins in comparison with conventional resins [38,39]. For instance, in 2018, Tahayeri et al. [38] compared the mechanical properties of 3D-printed interim resins (NextDent C&B, Vertex Dental, The Netherlands) manufactured with a stereolithography 3D printer (FormLabs1+, Formlabs Inc., Somerville, MA, USA) against conventionally cured interim resins (Integrity, Dentsply, CA, USA and Jet, Lang Dental Inc., Wheeling, IL, USA). The results of this paper [38] corroborated the data obtained in our study, highlighting that the tested 3D-printed provisional restorative material had sufficient mechanical properties for intraoral use, despite the limited accuracy of the 3D printing system of choice. Similarly, Simoneti et al. (2020) [39] evaluated the properties of interim fixed restorations fabricated by 3D printing employing different technologies (laser stereolithography (SLA) technology and selective laser sintering (SLS)) against restorations obtained by conventional techniques from self-cured acrylic resin and bis-acryl resin. The resins were assessed for fracture resistance (three-point bending flexural test), surface roughness, Vickers microhardness, fatigue, and biofilm formation. The SLS resin obtained favorable results for the Vickers microhardness and biofilm formation and had a higher maximum flexural strength and peak stress in the load-to-fracture tests and fatigue test, in comparison with the self-cured acrylic resin and bis-acryl resin, while the SLA resin obtained good results for surface roughness and biofilm formation.

At the same time, recent scientific studies [40,41,42,43] have focused on analyzing the mechanical behavior of interim fixed prosthetic materials obtained using different additive manufacturing technologies compared to milled resins or conventional resins; their results underline the superiority of the mechanical behavior of the interim prosthetic materials obtained by CAD/CAM technology over the behavior of conventional materials [40,41,42,43]. Reeponmaha et al. (2020) [40] showed that CAD/CAM manufactured interim crowns (obtained from a milled resin (Brylic Solid, Sage bioceramics, WA, USA) and from a 3D-printed resin (Freeprint Temp, Detax GmbH, Ettlingen, Germany)) and conventionally fabricated bis-acryl interim crowns (Protemp 4, 3M ESPE, Seefeld, Germany) demonstrated significantly higher fracture strength compared to conventionally fabricated monomethacrylate resin crowns (Unifast Trad, GC chemicals, Tokyo, Japan) after applying the aging regimen. Therefore, the authors suggested that CAD/CAM milling and 3D printing of interim prostheses could represent viable solutions for long term provisionalization. Another study [41] showed that interim crowns obtained via indirect techniques (CAD/CAM milling and 3D printing) obtained a higher score for fractured resistance force compared to interim crowns obtained via direct techniques from composite resins. Nevertheless, the authors of the study suggested that direct techniques can be beneficial since interim composite resin crowns are easier to repair. In line with the above-mentioned studies, Park et al. [42] reported that 3D-printed resin samples obtained via DLP (NextDent C&B, NextDent by 3D Systems, Vertex B.V., Soesterberg, The Netherlands) and via SLA (Temporary CB, Formlabs, Formlabs Inc., Somerville, MA, USA) demonstrated significantly higher flexural strengths in comparison with self-curing samples (Jet Tooth ShadeTM Powder; Lang Dental Co., Chicago, IL, USA); however, when compared to milled resin samples (ViPi block monocolor; VIPI Co., São Paulo, Brazil), no significant differences were observed in the flexural strength. Moreover, a relatively recent study, performed in 2021 [43], underlined that the tested 3D-printed material (Freeprint Temp; Detax GmbH & Co, Ettlingen, Germany) showed a similar flexural strength but better microhardness when compared to CAD/CAM milled samples (Ceramill Temp, shade A1, AmannGirrbach, AG, Koblach, Austria) and auto-polymerized conventionally fabricated samples (Jet Tooth Shade^TM^ Self-Curing Acrylic Resin, 6/1 Kit-Lang Dental Manufacturing Co., Inc. Illinois, IL, USA). Consequently, the authors concluded that 3D printing technology is potentially applicable for fabricating interim resin prostheses for clinical use [43].

On the other hand, the comparison of the results from various available scientific studies, in terms of mechanical behavior or the accuracy of additive manufactured interim prosthetic resins, is considered particularly challenging [20,23]. This is due to the fact that the properties of 3D-printed interim prostheses can be influenced by hydrothermal aging effects and various technical printing protocols or printing parameters, such as the printing speed/layer thickness and number of printed layers [44,45]; material shrinkage rate; position and angle of the restoration on the printing platform/build orientation [44,46,47,48,49]; amount of supportive material and post-processing procedures [46,47,48]; and type of design software [14,15,46,47,50,51]. In addition to the results of the previously presented studies, a recent paper [52] pointed out that screw-retained implant-supported interim crowns obtained via subtractive technology demonstrated a higher fracture resistance than those fabricated via vat-polymerized DLP additive technology methods. However, this study [52] compared 3D-printed implant-supported interim crowns with milled crowns, and conventional interim crowns were not subjected to the investigation.

It is possible that the mechanical performances achieved in our study by the 3D-printed resin in the 3DCS set (NextDent C&B MFH, NextDent by 3D Systems, Vertex B.V., Soesterberg, The Netherlands) were generated by the fact that the material was integrated in a manufacturing control system. The 3DOS resin (HARZ Labs Dental Sand, HARZ Labs, Riga, Latvia), by contrast, was processed in an open workflow. It is known that CAD/CAM systems can be classified into open and closed systems [53,54]. Closed systems are usually restricted to a single supplier and recommend a specific workflow, thus preventing interactions with other systems and offering a controllable, more predictable result [53,54]. Technologically closed systems provide an integrated workflow with coordinated phases: from computerized design to actual printing, then to post-polymerization [53,54]. On the other hand, in open systems, all the CAD/CAM components, including data acquisition, design (CAD software), and manufacture (CAM system), can be provided by different suppliers [53,55]. An open system allows the transfer of data to various design and restoration manufacturing devices; therefore, dental practitioners could combine features offered by different manufacturers in order to better meet the needs of their clinical practice [53,55]. Dental laboratories and dental practices (chairside digital workflow) sometimes tend to use 3D printers as a part of open access systems, as such systems offer greater flexibility and allow the usage of an extensive range of printing materials [56]. In the context of the rapid development of 3D printing applications in dentistry [57], the transition of CAD/CAM technologies used in dentistry from closed to open access systems could also meet the interest in the development of 3D printing in dentistry [58]. Moreover, establishing a “collaborative capacity network” in the dental market in the future would prove to be effective [58].

Although manufacture/fabrication by additive technologies can be recommended for both tooth- and implant-supported interim fixed prostheses [59], new interim 3D printing dental materials need to be further investigated in regard to their accuracy, reproducibility, mechanical properties, and long-term behavior [59,60]. Dentistry is continuously evolving, embracing the use of the latest technologies [60], and additive manufacturing is an emerging methodology that provides a cost-effective solution in dentistry [59]. Due to their rapid growth, there is a reduced amount of data regarding the mechanical properties of 3D-printed interim prosthetic materials [59]. Nevertheless, additive manufacturing (AM) proves a much higher potential for customization and complex geometries when compared to conventional manufacturing [61].

Regarding the results obtained in this study for the tested interim conventional acrylic resins (auto-polymerized and pressure/heat-polymerized acrylic resin), some remarks should be noted, as follows. The proper mechanical behavior of fixed interim prostheses is essential for protecting the oral structures and promoting adequate function and aesthetics for a limited period of time [62,63]. Interim treatment should satisfy the criteria of strength, marginal adaptation, and longevity [62]. Material selection has an important role in the performance of interim fixed dental prostheses [64]. As stated earlier in this paper, the most common materials used to fabricate interim fixed prostheses have traditionally been conventional resins (materials based on monomethacrylates or acrylic resins and materials based on dimethacrylates or bis-acryl/composite resins) [1,3,9,11,12,19]. However, the scientific literature indicates that the failure of interim restorations and other deficiencies are encountered by clinicians on a daily basis, mostly in the case of conventional, custom-fabricated interim restorations [11,62,63,64,65,66]. In this regard, Peñate et al. (2015) [65] showed that the fracture strength of directly (conventional) fabricated interim fixed dental prostheses was lower than of those fabricated by means of CAD/CAM technology [65]. Conversely, Digholkar et al. (2016) [67] pointed out that conventional heat-cured PMMA and milled PMMA had higher flexural strengths (95.58 MPa and 104.20 MPa, respectively) than 3D-printed resins (79.54 MPa). Concerning the mechanical behavior of conventional interim resins, Yilmaz et al. [66] pointed out that polycarbonate crowns demonstrated the highest values for fracture resistance (585.0 ± 42.778 N) when compared to crowns fabricated using bis-acryl composite (380 N), auto-polymerizing PMMA resin (448.3 N), and heat-polymerized PMMA resin (253.3 N). It is also worth mentioning that in this study [66], the heat-polymerized resin demonstrated a lower strength value than the auto-polymerizing acrylic resin, and a statistically significant difference between them was observed (*p* < 0.05). The authors suggested that one of the possible reasons for this result could be the strong plasticizer effect of the residual monomer within the auto-polymerizing acrylic resin. Thus, fractures could occur after the load was applied to the heat-polymerized acrylic resins, in which a rigid structure is formed. In the case of auto-polymerizing acrylic resins, plastic deformation might occur first and immediately thereafter the appearance of failures in the form of fractures. Another possible reason mentioned by the authors [66] for the higher values of fracture resistance in the auto-polymerizing acrylic resin when compared to the heat-polymerized PMMA resin may have been the addition of glycol dimethacrylate to the auto-polymerizing material, which provides molecular cross-linking. Another study [64] showed that dual-polymerizing bis-acrylic composite resin demonstrated higher stiffness and material strength and provided higher structural strength than auto-polymerizing bis-acrylic composite resin [64]. Over the years, different attempts have been made to enhance the mechanical properties of conventional acrylic resins. As an example, Berge et al. [68] reported that the effect of curing methods on the number and size of pores occurring in resin materials varied with the type of material: the highest numbers of pores occurred in unfilled heat-polymerized acrylic resin and in light-activated resin, whereas the lowest numbers were found in heat-polymerized microfilled acrylic resin. Murakami et al. [63] also showed that polymerization under a high pressure of 500 MPa by means of an isostatic pressurization machine at 70 °C for 24 h increased the toughness of an experimental PMMA resin (toughness was described as being the amount of elastic and plastic deformation energy required to fracture the acrylic resin specimens) [63]. Nevertheless, a recent study (Bauer et al. (2021) [69]) provided encouraging results, demonstrating that interim materials such as milled PMMA and di-methacrylate resins were sufficiently fracture resistant for the fabrication of interim implant-supported anterior fixed partial dentures and were expected to survive between 6 months and 2 years before failure. However, in this study [69], the tested conventional resin was a di-methacrylate resin, and auto-polymerized or pressure/heat-polymerized acrylic resins were not included. Given the previously described context, which indicates a relative heterogeneity in the mechanical behavior of interim conventional acrylic resins, and the results obtained in our study, we believe that conventional manufacturing and its repeatability and reproducibility could be strongly dependent upon the dental technician’s skills. Furthermore, conventional fabrication procedures could generate inhomogeneous samples that exhibit unfavorable mechanical behavior when compared to samples obtained via the additive method. In our study, the tested pressure/heat-polymerized acrylic resin (CHP) was characterized as being fragile, inhomogenous, and failing without absorbing energy when compared to the other resins included in the experiments. The topics at hand require continuous scientific evaluation.

Knowledge surrounding the mechanical properties of printable biomaterials is a prerequisite for achieving optimal performance in each clinical case when using 3D printing technologies in dentistry, including the design and manufacturing of oral appliances [59,61]. The present work confirms the data found in the dental literature and could contribute to guiding dental practitioners in properly selecting materials designated for interim fixed dental prostheses for each separate clinical case. Nevertheless, the limited number of investigated materials and mechanical tests performed represent two limitations of the present study. Furthermore, the experimental design of this in vitro study still has limitations in accurately expanding the obtained results to real clinical conditions; the mechanical tests that were used in our study differ from a cyclical load that would have simulated the masticatory load in a better way. It is our intention to include more prosthetic materials—and also the assessment of other properties of prosthetic materials, such as biocompatibility, accuracy, tensile strength, fatigue strength, repairability, or color stability—in future in vitro studies and clinical trials.

## 5. Conclusions

The present in vitro study evaluated the mechanical behavior under compression and bending of certain materials used for obtaining fixed prosthetic provisional restorations: two types of 3D-printed resins, an auto-polymerized conventional acrylic resin, and a pressure/heat-cured acrylic resin. Following the assessment of the results obtained, and given the limitations of the present study, we reached the following conclusions:

1. The tested 3D-printed interim resins obtained better results than the conventional resins in both the compression and flexure tests;
2. The 3D-printed resin coded 3DCS demonstrated the best mechanical behavior during the performed tests;
3. From a structural point of view, the tested 3D-printed materials presented a better homogeneity than the conventional materials.

## Figures and Tables

**Table 1:** Summary of information regarding the tested interim prosthetic materials.

Material Name	Manufacturer	Material (Resin) Type	Chemical Composition
NextDent C&B MFH	NextDent by 3D Systems, Vertex B.V., Soesterberg, The Netherlands	3D-printed resin/DLP Microfilled hybrid material PMMA (poly (methyl methacrylate))-based resin Lot: NDCMN101000_1	NP ^a^
HARZ Labs Dental Sand	HARZ Labs, Riga, Latvia	3D-printed resin/LCD PMMA (poly (methyl methacrylate))-like Microfilled composite resin Lot: 4603740518543	NP ^a^
Duracyl	SpofaDental a.s., Jicin, Czech Republic, KaVo Kerr Group	Auto-polymerized acrylic resin PMMA (poly (methyl methacrylate))-based Lot: 7169865	Powder: poly (methyl methacrylate), BPO (benzoyl peroxide), pigments, initiator, plasticizers, gelatin, talc, mineral and organic dyes Liquid: methyl methacrylate, DMTP (dimethyl terephthalate), polymerization activator
Superpont C+B	SpofaDental a.s., Jicin, Czech Republic, KaVo Kerr Group	Pressure/heat-cured acrylic resin PMMA (poly (methyl methacrylate))-based Lot: 6766480	- Powder: poly (methyl methacrylate), BPO (benzoyl peroxide), pigments, initiator, plasticizers, gelatin, talc, mineral and organic dyes Liquid: methyl methacrylate, triethylene glycol dimethacrylate, hydroquinone (polymerization inhibitor)

^a^ Abbreviations: NP—not provided.

**Table 2:** Mechanical characteristics of interim prosthetic resin samples in compression.

Sample Set	Elastic Modulus [MPa]	Elastic Limit [MPa]	Yield Strength [MPa]	Shortening [%]	Barreling [%]
CAP	1315 ± 186	43 ± 10	73 ± 8	16 ± 1.0	34 ± 5.0
CHP	1346 ± 379	46 ± 9	64 ± 15	NA	NA
3DOS	2419 ± 16	63 ± 3	97 ± 2	11 ± 1.0	19 ± 1.6
3DCS	2615 ± 183	75 ± 3	110 ± 9	13 ± 0.7	23 ± 1.0

**Table 3:** Mechanical characteristics of interim prosthetic resin samples in bending.

Sample Set	Elastic Modulus [MPa]	Bending Strength [MPa]	Strain at Failure [%]
CAP	4124 ± 333	88 ± 10	1.5 ± 0.01
CHP	4022 ± 1167	76 ± 7	1.1 ± 0.33
3DOS	6329 ± 79	141 ± 17	1.34 ± 0.20
3DCS	6402 ± 69	143 ± 15	1.38 ± 0.19

## Data Availability

The data are contained within the article.
